# Biohydrogenation of Linoleic Acid by Lactic Acid Bacteria for the Production of Functional Cultured Dairy Products: A Review

**DOI:** 10.3390/foods5010013

**Published:** 2016-02-23

**Authors:** Gabriela Christina Kuhl, Juliano De Dea Lindner

**Affiliations:** Food Science and Technology Department, Federal University of Santa Catarina, Florianópolis 88034-001, Brazil; gabrielac.kuhl@hotmail.com

**Keywords:** functional food, conjugated linoleic acid, biohydrogenation, lactic acid bacteria, dairy products

## Abstract

Conjugated linoleic acid (CLA) isomers have attracted significant attention due to their important physiological properties, which have been observed in humans. Many lactic acid bacteria (LAB) demonstrate the ability to produce CLA isomers (C18:2 *cis*-9, *trans*-11 and C18:2 *trans*-10, *cis*-12) from the linoleic acid (LA) present in milk or in synthetic media. CLA isomers can be synthesized *in vitro* by LAB using vegetable oils rich in LA. The aim of this review is to present an update on the studies that have been conducted on the production of CLA isomers from LA mainly by LAB and of the factors that influence this conversion (source and concentration of LA and fermentation conditions). In addition, this review presents the relationship between the consumption of CLA isomers and their health benefits in humans such as anti-atherosclerosis and anti-carcinogenic effects. There is considerable variation between the studies concerning the beneficial effects of CLA in animal models, which have not been reflected in human studies. This can be attributed to the differences in the doses of CLA isomers used and to the different sources of CLA. Furthermore, the regulatory and scientific information classifying the physiological properties of CLA, which serve as support for the claims of its potential as a functional ingredient, are presented. More research is needed to determine whether CLA production by LAB can be enhanced and to determine the optimal requirements for these microbial cultures. Furthermore, safety and efficacy of CLA consumption have to be investigated in the future.

## 1. Introduction

The pharmaceutical industry offers a wide range of drugs to cure many illnesses, and the trend is shifting towards the use of some diets or food bioactive compounds that could potentially prevent some chronic diseases, if they are taken together with a healthy lifestyle, as an alternative or compliment to medicine.

The consumption of certain fruits, vegetables, herbs and dairy products, especially the fermented ones, has been recommended as part of a healthy diet to provide essential nutrients and ease or cure many chronic diseases [1]. In the 1980s, the term “functional foods” (FOSHU—foods for specified health use) was introduced in Japan to characterize a new understanding of foods that included their medicinal properties as a way to decrease the costs to the states with regard to the population’s healthcare. Functional foods are those that, besides contributing to nutrient intake, contain substances that may be considered biologically active (promote biomodulation) and, consequently, could produce health benefits [2].

In recent years, dairy products and certain milk-derived components (e.g., whey proteins) have been highlighted as a category of functional foods. Significant functional dairy foods include bioactive milk peptides, products of fermented lactic acid bacteria (LAB), probiotics and some fatty acids (FAs). Conjugated linoleic acid (CLA) isomers are the most important bioactive component in milk fat and have received particular attention in recent years [3,4].

According to Brazilian law, the functional property of a food is related to the metabolic or physiological role that the nutrient or non-nutrient components plays in the growth, development, maintenance and other normal functions of the human body [5]. The Ministry of Health, through the National Health Surveillance Agency (ANVISA), regulates functional foods through Resolutions 16, 17 and 19 [6,7,8]. Commercialization of CLA isomers are forbidden in Brazil by ANVISA through Technical Report No. 23, 2007, which claims that current scientific evidence does not demonstrate the safety and efficacy of their use [9].

According to the US Food and Drug Administration (FDA), CLA isomers have been deemed safe for human consumption since July 11, 2008 and have received the designation of Generally Recognized As Safe (GRAS) under the GRN 00232 code because of scientific claims of their capacity for inflammatory suppression in the bowel and the improvement of specific responses to antigens by T cells against viral and bacterial pathogens [10].

Following a request from the European Commission (EC), the Panel on Dietetic Products, Nutrition and Allergies from European Food Safety Authority (EFSA) was asked to provide a scientific opinion on a list of health claims under the terms of article 13 of EC Regulation No. 1924/2006: “This opinion addressed the scientific basis of the health claims in relation to CLA isomers and their contribution to the maintenance or realization of a normal body weight, increased lean body mass, increased insulin sensitivity, and protection of DNA, proteins and lipids against oxidative damage, as well as their contribution to the immune system via the stimulation of the production of protective antibodies in response to vaccination. The scientific rationale for this opinion was based on the information provided by the Member States in the consolidated list of article 13 on the health claims and references that the EFSA has received from Member States or directly from stakeholders. The food constituent that is the object of these health claims is an equimolar mixture of isomers C18:2 *cis*-9, *trans*-11 and C18:2 *trans*-10, *cis*-12. The Panel considered the equimolar mixture of isomers to be sufficiently characterized” [11].

As for the Canadian Food and Drugs Act, based on its definition of food and medicine, it limits health claims related to food, food ingredients and natural health products (NHPs). In recent decades, scientific research has led to a large body of information demonstrating the health benefits of many foods and NHP ingredients. Canadian health authorities have recognized the limitations of the current regulation and have begun to develop regulations related to the support of the health claims made for functional foods and NHPs, including those containing CLA isomers. For nutritional labelling purposes, mixtures of the two most prominent CLA isomers (C18:2 *cis*-9, *trans*-11 and C18:2 *trans*-10, *cis*-12) are not considered *trans* fats. Therefore, it is not required to declare the content of CLA isomers in the nutritional information about *trans* fats on the label [12].

CLA isomers attracted considerable attention after several *in vivo* experiments demonstrated the important physiological properties of these molecules. After discovering the CLA isomer content present in dairy products and their benefits to human health, including anti-cancer [13], anti-diabetic [14], anti-atherosclerotic [15], and anti-osteoporosis [16] properties, as well as the prevention of increases in body fat [17] and their function as a stimulator of the immune system [18], these molecules have become an object of study for applications in food production.

The daily intake of CLA isomers has been estimated in different countries through different techniques, such as the establishment of nationwide food surveys, food frequency questionnaires, dietary estimates and seven-day food records. Daily consumption is very low and ranges from 0 to 500 mg for people in most of the countries surveyed [19]. Dietary supplements of CLA isomers are mainly marketed in North America and European countries as gel capsules that provide *cis*-9, *trans*-11 and *trans*-10, *cis*-12 isomers in different proportions.

An alternative to dietary supplements, which are usually less preferred than the supply of nutrients in foods, is the direct increase of the CLA content of food. This is a practical approach because there are no changes in the diet or its components and no increase in the daily intake of cholesterol and saturated fat. The management of the CLA content of food, such as dairy products, provides an important way to increase their nutritional and functional value and may have a considerable marketing effect by adding value to traditional products. An increase in the concentration of CLA isomers in ruminant milk is an interesting option because of their important presence in this food matrix. The natural synthesis of these molecules is from the metabolism of ruminal microorganisms and they are present in ruminant tissues [20]. In addition to ruminal bacteria, many food-grade microorganisms are able to produce CLA isomers from the LA in milk [21,22,23,24,25,26]. Because the natural concentrations of CLA isomers in milk products are relatively low, to exert their health benefits, GRAS bacteria with linoleate isomerase (a strain-dependent enzyme responsible for CLA conversion) activity could be used as a microbial culture to develop functional, fermented dairy products with increased levels of CLA [27].

In this review, we summarize the most important contributions to the synthesis of CLA isomers by LAB and the factors that influence this production. In addition, we summarize the studies that have been conducted on the health benefits of CLA and present the regulatory and scientific information relating to the classification of the physiological properties of CLA, which provide support for its use in functional foods.

## 2. Methods

In this systematic review, we did not use statistical techniques (meta-analysis) to combine results of the eligible studies. We conducted an extensive bibliographical search using free search engines (PubMed and Web of Science) that access references and abstract databases on food science. The following key terms were used in our searches: conjugated linoleic acid, biohydrogenation of linoleic acid, conjugated linoleic acid conversion by lactic acid bacteria, and conjugated linoleic acid health benefits. The titles and abstracts of the articles found in the search were checked against the overall criteria for eligibility and relevance, which were as follows: the year of publication, the relevance of the research question, and the citation index.

## 3. Literature Review

### 3.1. CLA Isomers

*Trans* fatty acid (FA) isomers are rarely produced in nature. Thus, the major source of these *trans* FAs in the human diet is the industrial partial hydrogenation of vegetable oils. However, small quantities of these compounds are also formed in the rumen of polygastric animals via the bacterial biohydrogenation of unsaturated FA, which means they are mostly found in the milk and meat of ruminants [28,29,30].

The most abundant FAs, with a system of conjugated double bonds, are the isomers of LA known as CLA. The abbreviation CLA is used to describe the family of isomers of octadecadienoic acid (18:2) which have a pair of conjugated double bonds along the alkyl chain. Theoretically, the double bonds can exist at any location between carbons C_2_ to C_18_ to produce numerous structural isomers [31]. There are 28 possible isomers of CLA that differ in the position (7 and 9, 8 and 10, 9 and 11, 10 and 12, 11 and 13; based on the method used for counting the carboxyl group) and the configuration (*cis* or *trans*) of the double bonds [32].

### 3.2. Synthesis of CLA Isomers by Biohydrogenation

In recent decades, studies have demonstrated the biological significance of the CLA isomers. Meat and milk from ruminants and their products are the natural dietary sources of CLA. In the raw materials, the predominant isomer is C18:2 *cis*-9, *trans*-11, which accounts for over 75% of the total CLA. The C18:2 *cis*-9, *trans*-11 isomer is produced from LA as an intermediate in biohydrogenation by ruminal bacteria [31,33,34].

The lipids inside the rumen undergo transformation by microbial lipases in a process called lipolysis, which is considered to be the prerequisite for biohydrogenation. Microbial lipases hydrolyze ester linkages in the lipid complex, thereby forming free FAs. With the oxidation of the triacylglycerols to glycerol and free FAs, the synthesis of CLA begins, specifically, with the isomerization of unsaturated FAs and further biohydrogenation by ruminal bacteria [35]. The degree of lipolysis and biohydrogenation depend on the type and amount of fat received by the rumen and the ruminal pH [36]. The biohydrogenation is actually considered to be detoxification mechanism [21]. To minimize the toxic effects of LA on ruminal fermentation, ruminal bacteria uses linoleate isomerase to convert it to CLA. This mechanism may be the key to finding methods to manipulate the biohydrogenation in a predictable manner [36,37].

### 3.3. Conversion of CLA Isomers by LAB

The discovery of the production of CLA isomers by ruminal bacteria led to the speculation that other microorganisms may also be able to synthesize these metabolites [38]. Several studies have reported the production of CLA isomers during lactic fermentation by LAB [21,22,39]. According to these studies, some strains of bacteria were found to be able to change the FA profile of milk in addition to producing functional FA during fermentation as a result of bacterial growth and metabolism. There are important factors that can increase the production of CLA isomers by LAB. Among them, the authors describe variables such as the fermentation time and temperature, the protein concentration and the oil substrate (LA supply) [40]. Some authors have invested in strategies to improve the conversion process (Table 1).

According to [22] an LA source must be added to the medium to enhance CLA isomer formation by LAB strains. However, [42] presented 20 LAB strains that are able to produce CLA in milk (0.43–1.12 g of FA) without any substrate addition. Thus, some authors have investigated the factors affecting the CLA content during milk fermentation instead of investing in strategies to improve the conversion process.

In the work of Jiang, *et al.* [21], the abilities of 19 different strains commonly used as dairy starter cultures to produce CLA (C18:2 *cis*-9, *trans*-11 isomer represented more than 70% of the total CLA formed) from free LA were determined. They observed that most strains were inhibited at an LA concentration of 25 µg/mL. Among the CLA-producing strains, a positive correlation between CLA formation and the ability to tolerate free LA was observed, which suggests that the conversion of free LA to CLA might function as a detoxification mechanism. Furthermore, the authors concluded that each strain requires different treatment when exposed to certain conditions, such as LA concentration, temperature and fermentation time.

Fourteen LAB strains were screened for their ability to produce CLA using sunflower oil as a substrate. *Lc. lactis* showed the highest production of CLA from sunflower oil (4 mg/g fat or more) and was selected for further studies to identify the factors and processes responsible for increasing CLA in fermented milk [23]. The highest CLA formation (8.5 mg/g fat) was detected with sunflower oil (0.2 g/L) in combination with dry milk powder (6%) and glucose (0.3%) and 12 h incubation. The results also demonstrated that CLA formation in fermented milk can be affected by several factors, including the microbial strain, number of cells, incubation time at neutral pH and substrate concentration.

LAB strains (*Lactobacillus* and *Streptococcus*) and *Bifidobacteria* were inoculated in MRS (Man Rogosa Sharpe) broth with added LA (200 µg/mL) to evaluate their CLA-producing capacity [24]. After initial screening, four strains that demonstrated the greatest ability to produce CLA from LA were selected to test the production of CLA in buffalo milk at different LA concentrations (200–1000 µg/mL). CLA production was shown to be strain dependent at different LA levels. *Lb. casei* showed the highest CLA production level (175.2 µg/mL) at 400 µg/mL LA, while *Lb. rhamnosus* and *Bifidobacterium bifidum* produced the most CLA (190.2 and 90 µg/mL, respectively) at 200 µg/mL LA and, finally, *St. thermophilus* showed the highest CLA production (198.6 µg/mL) at 800 µg/mL LA.

Different LAB strains (*Lb. acidophilus, Lb. lactis, Lc. cremoris, Lc. lactis, Lb. bulgaricus* and *St. thermophilus*) were inoculated in a growth medium containing 12% milk powder and LA concentrations of 1000 or 5000 μg/mL and were incubated for 0, 24 or 48 h. CLA (C18:2 *cis*-9, *trans*-11 isomer) production was significantly changed in *Lb. bulgaricus* (from 86 to 66 μg/mL) and *St. thermophilus* (from 82.5 to 57 μg/mL) cultures by prolonging the incubation time from 24 h to 48 h and with 5000 μg/mL LA [22]. However, another study on *Lb. bulgaricus* was not able to produce CLA in MRS broth supplemented with 100 or 200 µg/mL of LA at 37°C and incubation times of 12 or 24 h [23,24].

Recent studies have evaluated the independent variables for milk fermentation to determine the best conditions to increase the CLA content of dairy products [25,26]. Khosravi-Darani, *et al.* [25] used a Plackett-Burman design to study the effects of seven variables (whey powder‚ grape seed oil, temperature, pH, incubation time and inoculum age and size) on CLA production by different probiotic strains (*Lb. acidophilus*, *B. bifidum* and *P. freudenreichii*). The highest amount of CLA (11.03 mg/g fat) was obtained with the following combination of factors: 4% (*w*/*v*) whey powder, 4% (*v*/*v*) grape seed oil, pH 6.0, inoculum size of 0.8% (*v*/*v*), inoculum age of 36 h, a 35°C fermentation temperature, and an incubation time of 27 h. The ability of different *Lactobacillus* strains to produce CLA from LA was studied by A., *et al.* [26]. The experiments revealed that *Lb. plantarum* had the highest CLA producing (95.25 μg/mL) potential of the strains screened. A response surface methodology was applied to investigate the effects of three independent variables (LA, yeast extract concentration and inoculum size) on CLA formation. The optimum conditions to improve the CLA production (240.69 μg/mL) were obtained by using 3 mg/mL of LA, 4 g/L of yeast extract and an inoculum size of 4% (*v*/*v*).

Xu, *et al.* [41] assayed several lactic bacteria inoculums to obtain CLA, in a model system containing milk hydrolyzed soybean oil. *Propionibacterium freudenreichii* ssp. *shermanii* was the higher CLA (C18:2 *cis*-9, *trans*-11) producer (1.45 mg/g of fat) after fermentation time. Later, [43] evaluated the formation of CLA in fermented milk products from hydrolyzed soy oil. The authors tested probiotic strains individually or in co-culture with traditional yogurt cultures (1:1 ratio of *Lb. bulgaricus* and *St. thermophilus*). In the presence of yogurt cultures, a slightly higher content of CLA was found after 14 days of storage. *Lb. rhamnosus* in co-culture produced the highest levels of C18:2 *cis*-9, *trans*-11 (0.97 mg/g lipid) and C18:2 *trans*-10, *cis*-12 (0.71 mg/g lipid) among the probiotic bacteria assayed. In a similar study, cow and sheep milk yogurts were investigated for changes in CLA (C18:2 *cis*-9, *trans*-11 isomer) concentration during 14 days of storage. Refrigerated storage resulted in a significant decrease in the CLA in cow milk yogurts and in a significant increase in the CLA in sheep milk yogurts, suggesting that CLA content was influenced by the origin of milk [44].

### 3.4. CLA Isomers Health Benefits

Studies have been conducted to identify CLA consumption in many countries. Estimates range from 0.2 to 1.5 g/person/day and appear to depend on sex and food sources (from animal or vegetable origins) [31,45,46,47]. For example, the Brazilian population was found to have one of the lowest levels of dietary CLA intake reported (approximately 36 mg/day) [48].

Several experiments have been initiated to determine the physical, biochemical and physiological properties of CLA isomers. Research indicates that these isomeric conjugated FAs have potent biological activities that have been suggested to benefit human health [49] (Table 2).

According to Ip, *et al.* [50], supplementation of rat diets with 0.5% C18:2 *cis*-9, *trans-*11 isomer was sufficient to produce a significant reduction in the development a mammary tumors. C18:2 *cis*-9, *trans*-11 may be more effective than any other FA in tumor modulation. However, Ip, *et al.* [52] observed a maximum antioxidant activity of CLA at a 0.25% daily dose in rat diets, suggesting a discrepancy between the doses needed for effective antioxidant and anti-carcinogenic activities.

Another study carried out by [51] suggested a daily intake of 3 g/day of CLA (a mixture of total isomers with the following composition: C18:2 *cis*-9*, trans*-11 and *trans*-9, *cis*-11, 43.3%; C18:2 *trans*-10, *cis*-12, 45.3%; C18:2 *cis*-9, *cis*-11, 1.9%; C18:2 *cis*-10, *cis*-12, 1.4%; C18:2 *trans*-9, *trans*-11 and *trans*-10, *trans*-12, 2.6%) to achieve the inhibitory effects on carcinogenesis in mammary gland rats. Animals weighing 350 g were fed a diet with 0.1% CLA (approximately 0.015 g of CLA/day), which is equivalent to a daily dose of 3 g of CLA for a 70 kg person. This is slightly higher than the estimated average consumption.

The molecular mechanisms of the bioactivity of CLA have been attributed to its ability to modulate eicosanoid formation and gene expression. Very few clinical studies in humans have been conducted. Nevertheless, *in vitro* and *in vivo* studies have shown antioxidant and anticancer effects for the C18:2 *cis*-9, *trans*-11 isomer [28]. The inhibitory effects of CLA on different types of cancer, such as breast, skin, stomach, intestine and prostate cancer, have been consistently demonstrated in most studies, which have involved different animal models and human cell cultures [52,53].

Several studies reviewed by Belury, *et al.* [59] and Pariza, *et al.* [60] have demonstrated the ability of CLA (C18:2 *cis*-9, *trans*-11 and C18:2 *trans*-10, *cis*-12 isomers) to modulate the inflammatory response and restore insulin sensitivity in animals with type-II diabetes. Recently, it has been shown that the modulation of inflammatory responses by CLA (C18:2 *cis*-9, *trans*-11 isomer) seems to have a positive influence on biochemical markers associated with Alzheimer’s disease (AD). Barbosa, *et al.* [62] reported a reduction in the activity of different phospholipases (A_2_ (PLA_2_) subtypes) in the rat brain. PLA_2_is a family of hydrolytic enzymes that are important mediators in the transmission and processing of neuronal signals and in the regulation of other active products. Forty-eight animals were fed three different diets over a thirty-day period. The findings of the study suggest that a diet enriched with a 60% CLA supplement (30% of C18:2 *trans*-10, *cis*-12 and 30% of C18:2 *cis*-9, *trans*-11) may be a potential nutraceutic for the prevention and/or inhibition of the progression of Alzheimer’s disease. Gama, *et al.* [63] analyzed 30 rats fed with three different diets over four weeks. Treatments consisted of a CLA-free diet and two diets containing butter enriched with the C18:2 *cis*-9, *trans*-11 isomer (0.72 and 1.98 g/100 g of total fat). Compared with the effects of the control diet, the hippocampal activity of the PLA_2_ enzyme group was increased by diets enriched with CLA. These results suggest that milk products enriched with CLA may be helpful in AD therapy.

An *in vivo* study evaluated the effects of industrial *trans* FAs and CLA on inflammation and oxidative stress markers in 61 healthy adult subjects. The authors observed a beneficial effect on inflammatory markers and on one oxidative stress marker in volunteers who consumed a mixture of CLA isomers (80% C18:2 *cis*-9, *trans*-11 and 20% C18:2 *trans*-10, *cis*-12) in their diet [64].

In a recent study, hypertriacylglycerolmic (HHTg) rats were fed for two months with a diet supplemented with a 2% mixture of CLA isomers (1:1, C18:2 *cis*-9, *trans*-11:C18:2 *trans*-10, *cis*-12) or with sunflower oil (control group). CLA-treated rats showed a decrease in body and visceral adipose weight and a reduction in triacylglycerol accumulation in the serum liver, heart, muscle and aorta. In addition, compared with the effects in the control rats, CLA treatment resulted in an increase in insulin sensitivity. In this study, the authors concluded that CLA supplementation may protect against HHTg-induced dyslipidaemia, ectopic lipid deposition and insulin resistance [14].

De Guire, *et al.* [16] demonstrated an association between the CLA content of erythrocyte membranes (RBC) and body composition and bone mineral density. Fifty-four men (age 19–53 years) were tested in a cross-sectional study to examine the association between the C18:2 *cis*-9, *trans*-11 content of RBC membranes and body composition. The study used a dose-response trial and randomized the subjects to 1 of 3 groups: placebo, 1.5 g/day or 3.0 g/day of CLA over a 16-week period. Bone mineral density was significantly higher in the group receiving 3.0 g of CLA compared with that of the group receiving 1.5 g, whereas the placebo group was not different from any other group.

Studies on obese individuals have proposed that a dose of 3.0 to 4.2 g/day of CLA may result in a loss of body fat in humans [54,55,56,57,58,61]. However, although the C18:2 *trans*-10, *cis*-12 isomer has been associated with anti-adipogenic effects in humans, the results are less significant and more ambiguous compared with those observed in other animals [65]. Nevertheless, scientists researching this topic believe, when the body of evidence is considered as a whole, that C18:2 *trans*-10, *cis*-12 does have a beneficial effect on human body composition if it is consumed over time and that it does not appear to result in any adverse effects [57,66].

## 4. Conclusions

Milk and its individual components in their intact or hydrolyzed forms may be considered to be a complete nutritional food, and its bioactive compounds may be considered to be functional ingredients. With the growing number of consumers aware of the importance of the consumption of functional foods in maintaining health, the importance of dairy products as a relevant source of bioactive compounds is clear. The use of LAB and *Bifidobacteria* in the production of CLA isomers contributes to the increased functional potential of milk because the incorporation of these strains, among its other benefits, allows for an increase in the CLA isomer content of fermented dairy products. More research is needed to assess if CLA production by LAB can be enhanced and to determine the optimal requirements for these microbial cultures. Fermented milk products can be naturally enriched with CLA isomers, providing synchronization between diet and health and promoting new opportunities for the dairy market. The current state of our knowledge is limited with regard to the effects of CLA isomers in humans. Adverse effects still were not described about CLA consumption, other questions related to the safety and efficacy have to be answered such as for example, oxidative stress, insulin resistance and irritation of intestinal tract. Furthermore, there are considerable variations between the studies concerning the beneficial effects of CLA in animal models, which have not been reflected in human studies. There is need to conduct more human studies with large number of participants with and without comorbitities and higher CLA intake intervals.

## Figures and Tables

**Table 1:** Summary of the strategies to increase conjugated linoleic acid (CLA) conversion by bacteria. Total CLA ^a^; C18:2 *cis*-9 ^b^, *trans*-11 isomer; Represented form was not specified ^c^.

Microorganism	LA Source		Fermentation Time (h)	CLA Concentration	Ref.
	Oil Substrate	(µg/mL)			
*Lc. lactis*	Sunflower oil	200	12	8.5 mg/g fat ^a^	[23]
*Lb. casei*	LA	400	24	175.2 µg/mL fat ^a^	[24]
*Lb. rhamnosus*	LA	200	24	190.2 µg/mL fat ^a^	
*Bifidobacterium bifidum*	LA	200	24	90 µg/mL fat ^a^	
*St. thermophilus*	LA	800	24	198. µg/mL fat^a^	
*Lb. bulgaricus*	LA	5000	48	86 µg/mL medium ^b^	[22]
*Lb. acidophilus*	LA	1000	24	106.5 µg/mL medium ^b^	
*St. thermophilus*	LA	5000	48	82.5 µg/mL medium ^b^	
*Lb. plantarum*	LA	3000	24	240.69 µg/mL ^b,c^	[26]
*Lb. rhamnosus*	Hydrolyzed soy oil	1%	24	310 µg/g fat ^a^	[41]
*Lb. acidophilus*	Hydrolyzed soy oil	1%	24	450 µg/g fat ^a^	
*Lb. casei*	Hydrolyzed soy oil	1%	24	480 µg/g fat ^a^	
*Lb. plantarum*	Hydrolyzed soy oil	1%	24	510 µg/g fat ^a^	
*Bifidobacterium bifidum*	Hydrolyzed soy oil	1%	24	460 µg/g fat ^a^	
*Propionibacterium freudenerchii* ssp. *sherrmanii*	Hydrolyzed soy oil	1%	24	1450 µg/g fat ^a^	
Yogurt culture (*St. thermophilus + Lb. bulgaricus*, 1:1)	Hydrolyzed soy oil	1%	24	710 µg/g fat ^a^	
Yogurt starter culture + probiotic bacterias (*Lb. acidophilus, Bifidobacterium bifidum* and *Propionibacterium freudenerchii*)	Grape seed oil	4%	27	11.03 mg/g fat ^a^	[25]

**Table 2:** Biological activities of the CLA isomers.

CLA Isomer	Effects	Reference
C18:2 *cis*-9, *trans*-11; *trans*-10,*cis*-12 (1:1)	Decrease in breast tumor development	[50,51]
	Increased antioxidant activity	[50]
	Inhibitory effects on various types of cancer, such as breast, skin, stomach, intestinal, and ovarian	[52,53]
	Immunoregulatory effects	[18]
	Decrease in body fat mass	[17,54,55,56,57]
	Decreased sagittal abdominal diameter (cm)	[58]
	Modulation of the inflammatory response	[59,60]
	Increased lean body mass	[55,61]
	Decreased glucose levels	[14]
	Decrease in body fat mass in specific region (legs)	[61]
	Decreased waist-hip ratio	[61]
C18:2 *cis*-9, *trans*-11	Positive influence on biochemical markers associated with Alzheimer’s disease	[62,63]
	Anti-osteoporotic effects	[16]
	Anti-atherosclerotic effects	[15]
C18:2 *trans*-10, *cis-*12	Inhibitory effects on the development of ovarian cancer	[13]
	Inhibitory effects on prostate cancer	[53]
